# Validation of putative biomarkers of furan exposure through quantitative analysis of furan metabolites in urine of F344 rats exposed to stable isotope labeled furan

**DOI:** 10.1007/s00204-024-03722-5

**Published:** 2024-04-04

**Authors:** C. Kalisch, M. Reiter, M. Krieger, L. Wüst, C. Klotz, R. Dekant, D. W. Lachenmeier, O. Scherf-Clavel, A. Mally

**Affiliations:** 1 Department of Toxicology, University of Würzburg, Versbacher Str. 9, 97078 Würzburg, Germany; 2 Chemisches und Veterinäruntersuchungsamt Karlsruhe, Karlsruhe, Germany; 3 Institute for Pharmacy and Food Chemistry, University of Würzburg, Würzburg, Germany; 4 Department of Pharmacy, Ludwig-Maximilians-University of Munich, Munich, Germany

**Keywords:** Furan, Process-related food contaminant, Biomarker of exposure, Biomonitoring, Metabolism, Urinary metabolite excretion

## Abstract

Humans are chronically exposed to furan, a potent liver toxicant and carcinogen that occurs in a variety of heat-processed foods. Assessment of human exposure based on the furan content in foods is, however, subject to some uncertainty due to the high volatility of furan. Biomarker monitoring is thus considered an alternative or complementary approach to furan exposure assessment. Previous work suggested that urinary furan metabolites derived from the reaction of *cis*-2-butene-1,4-dial (BDA), the reactive intermediate of furan, with glutathione (GSH) or amino acids may serve as potential biomarkers of furan exposure. However, some metabolites were also reported to occur in urine of untreated animals, indicating either background contamination via animal feed or endogenous sources, which may limit their suitability as biomarkers of exposure. The overall aim of the present study was to accurately establish the correlation between external dose and concentration of furan metabolites in urine over time and to discriminate against endogenous formation and furan intake via feed. To this end, the furan metabolites GSH-BDA (*N*-[4-carboxy-4-(3-mercapto-1*H*-pyrrol-1-yl)-1-oxobutyl]-L-cysteinylglycine), NAcLys-BDA (*R*-2-(acetylamino)-6-(2,5-dihydro-2-oxo-1*H*-pyrrol-1-yl)-1-hexanoic acid), NAcCys-BDA-NAcLys (*N*-acetyl-S-[1-[5-(acetylamino)-5-carboxypentyl]-1*H*-pyrrol-3-yl]-L-cysteine) and NAcCys-BDA-NAcLys sulfoxide (*N*-acetyl-S-[1-[5-(acetylamino)-5-carboxypentyl]-1*H*-pyrrol-3-yl]-L-cysteine sulfoxide) were simultaneously analyzed by stable isotope dilution ESI–LC–MS/MS as unlabeled and [^13^C_4_]-furan dependent metabolites following oral administration of a single oral dose of isotopically labelled [^13^C_4_]-furan (0.1, 1, 10, 100 and 1000 µg/kg bw) to male and female F344/DuCrl rats. Although a linear correlation between urinary excretion of [^13^C_4_]-furan-dependent metabolites was observed, analysis of unlabeled NAcLys-BDA, NAcCys-BDA-NAcLys and NAcCys-BDA-NAcLys sulfoxide revealed substantial, fairly constant urinary background levels throughout the course of the study. Analysis of furan in animal feed excluded feed as a source for these background levels. GSH-BDA was identified as the only furan metabolite without background occurrence, suggesting that it may present a specific biomarker to monitor external furan exposure. Studies in humans are now needed to establish if analysis of urinary GSH-BDA may provide reliable exposure estimates.

**Supplementary Information:**

The online version contains supplementary material available at 10.1007/s00204-024-03722-5.

## Introduction

The process related food contaminant furan is formed in a range of heat-treated food items due to thermal degradation of natural food components. Relatively high levels of furan are found in coffee, coffee products and canned food, particularly processed baby food. Based on the concentration of furan in food and dietary surveys across European countries, highest dietary exposures to furan were estimated in infants and adults (European Food Safety Authority). In infants, mean and 95th percentile exposures ranged between 0.14 and 0.99 µg/kg bodyweight (bw) per day (minimum lower bound (LB) to maximum upper bound (UB)) and 0.19 to 1.82 µg/kg bw per day (minimum LB to maximum UB), respectively. Chronic dietary exposure in adults was estimated to range between 0.11 and 0.75 µg/kg bw per day (minimum LB to maximum UB) on average and between 0.20 and 1.22 µg/kg bw per day (minimum LB to maximum UB) in highly exposed consumers (95th percentile) (European Food Safety Authority 2017).

Based on the margin of exposure approach (MOE), EFSA concluded in its 2017 risk assessment that the current exposures to furan, a potent hepatotoxin and hepatocarcinogen, indicate a health concern (European Food Safety Authority 2017). For non-neoplastic effects, the MOEs calculated using a Benchmark dose lower confidence limit 10% (BMDL_10_) of 0.064 mg/kg bw per day for induction of cholangiofibrosis in male rats after 2 years as the point of departure were below 100 for some age groups, such as infants and toddlers, and were therefore considered to indicate a health concern. For neoplastic effects, EFSA selected a BMDL_10_ of 1.31 mg/kg bw per day for increased incidence of hepatocellular adenomas and carcinomas in female mice after 2 years as reference point. The calculated MOEs for neoplastic effects were smaller than 10,000 for most dietary surveys. However, EFSA emphasized the uncertainties in the present exposure assessment, which may lead to both under- and overestimation of dietary furan exposure (European Food Safety Authority 2017). In particular, furan formation during home cooking as well as evaporation losses of furan during standing of beverages after brewing (e.g., coffee) or reheating of commercially processed foods (e.g., ready-to-eat meals for infants) were not considered in the assessment (European Food Safety Authority 2017).

**Fig. 1:**
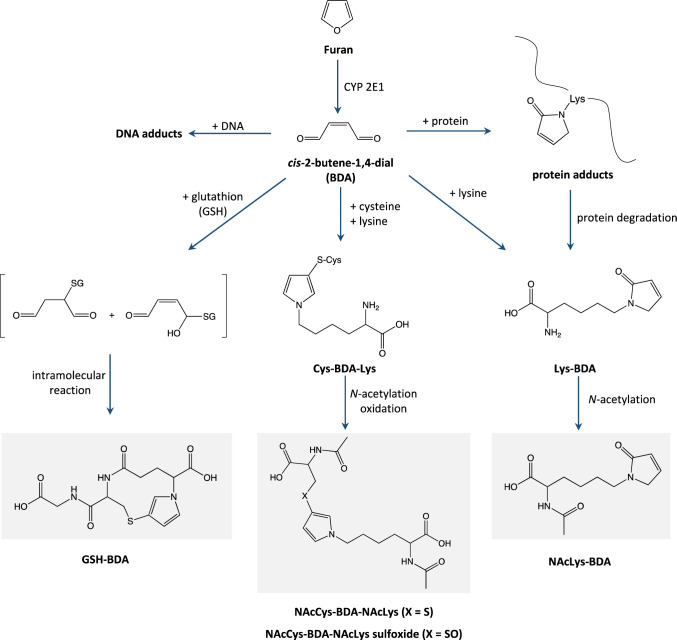
Metabolic pathways of furan and potential urinary biomarkers of furan exposure. Furan is predominantly metabolized by CYP 2E1, leading to the formation of the reactive dialdehyde *cis*-2-butene-1,4-dial (BDA). The reactivity of BDA towards cellular nucleophiles results in a broad spectrum of urinary metabolites: GSH-BDA (1), the product of the conjugation with glutathione and a subsequent intramolecular reaction; NAcLys-BDA (2), the adduct of BDA and lysine, followed by acetylation of the ⍺-amino group of lysine; NAcCys-BDA-NAcLys (3) and its corresponding sulfoxide (4), which derive from crosslinks of cysteine and lysine by BDA and subsequent *N*-acetylation and oxidation. Note that conjugation with GSH and cysteine can also occur in position 2 instead of 3 in the pyrrol ring

Biomonitoring has been suggested as an alternative approach to assess furan exposure via food. Several urinary metabolites derived from cytochrome P450 2E1 mediated bioactivation of furan to the highly reactive intermediate *cis*-2-butene-1,4-dial (BDA) and subsequent reaction of BDA with cellular nucleophiles such as free or protein-bound lysine and cysteine residues and glutathione (GSH) have been suggested as potential biomarkers of furan exposure (Kellert et al. 2008; Rietjens et al. 2018; Karlstetter and Mally 2020). These include* N*-[4-carboxy-4-(3-mercapto-1*H*-pyrrol-1-yl)-1-oxobutyl]-l-cysteinylglycine (GSH-BDA), a cyclic conjugate of BDA and GSH formed by an intramolecular reaction,* R*-2-(acetylamino)-6-(2,5-dihydro-2-oxo-1*H*-pyrrol-1-yl)-1-hexanoic acid (NAcLys-BDA) formed by reaction BDA with lysine and subsequent acetylation of the ⍺-amino group of lysine, as well as *N*-acetyl-S-[1-[5-(acetylamino)-5-carboxypentyl]-1*H*-pyrrol-3-yl]-l-cysteine (NAcCys-BDA-NAcLys) and* N*-acetyl-S-[1-[5-(acetylamino)-5-carboxypentyl]-1*H*-pyrrol-3-yl]-l-cysteine sulfoxide (NAcCys-BDA-NAcLys sulfoxide), which represent *N*-acetylated crosslinks of cysteine and lysine by BDA (Fig. 1). Previous quantitative analysis of urine collected from rats treated with furan at doses of 0.1, 0.5 and 2 mg/kg bw for 5 and 28 days demonstrated a linear correlation between external dose and urinary excretion of NAcLys-BDA and cyclic GSH-BDA, but also revealed substantial background levels of NAcLys-BDA in urine of untreated animals (Karlstetter and Mally 2020). This observation is consistent with qualitative data obtained by Kellert et al. who reported NAcLys-BDA and NAcCys-BDA-NAcLys in urine of untreated rats and considered animal feed or endogenous formation of furan or its metabolite BDA as potential sources for these background levels (Kellert et al. 2008).

In view of the background levels and limited quantitative data on furan metabolite excretion following oral intake, the aim of the present study was to further assess furan metabolites as potential biomarkers of furan exposure and to discriminate between the externally applied dose and background exposure through quantitative analysis of furan-dependent metabolites in urine of rats after a single oral dose of isotopically labeled furan. To this end, GSH-BDA, NAcLys-BDA, NAcCys-BDA-NAcLys and its corresponding sulfoxide were simultaneously quantified by stable isotope dilution ESI–LC–MS/MS as unlabeled and [^13^C_4_]-furan dependent metabolites in urine of male and female F344/DuCrl rats administered [^13^C_4_]-furan by oral gavage. To establish a correlation between external dose and urinary excretion of furan-derived metabolites, furan was administered across a wide dose range that includes doses relevant to human exposure (0, 0.1, 1, 10, 100 and 1000 µg/kg bw).

## Material and methods

### Chemicals and reagents

[^13^C_4_]-Furan and 2,5-diacetoxy-2,5-[^13^C_4_]-dihydrofuran were purchased from Cambridge Isotope Laboratories (Tewksbury, MA). 2,5-Diacetoxy-2,5-dihydrofuran was from Toronto Research Chemicals (Ontario, Canada). *N*-acetyl-l-lysine, *N*-acetyl-l-cysteine and [^13^C_2_]-*N*-acetyl-l-cysteine were purchased from Sigma–Aldrich (Schnelldorf, Germany). L-[^13^C_6_^15^N_2_]-lysine was obtained from Roth (Karlsruhe, Germany). [^13^C_2_^15^N_2_]-GSH-BDA was synthesized as previously described (Karlstetter and Mally 2020). HPLC grade acetonitrile, LC–MS grade methanol and LC–MS grade water were obtained from Roth (Karlsruhe, Germany). LC–MS grade formic acid was from Thermo Fischer Scientific (Waltham, MA). All other chemicals were from Merck (Darmstadt, Germany) or Roth (Karlsruhe, Germany), unless stated otherwise.

### Chemical syntheses

Reference compounds were prepared by reacting BDA with GSH or amino acid derivatives as previously described (Chen et al. 1997; Lu et al. 2009; Hamberger et al. 2010; Karlstetter and Mally 2020).

#### Preparation of *cis*-2-Butene-1,4-dial and *cis*-2-[^13^C_4_]-Butene-1,4-dial

##### *cis*-2-Butene-1,4-dial

*cis*-2-Butene-1,4-dial (BDA) was prepared by hydrolysis of 0.1 M 2,5-diacetoxy-2,5-dihydrofuran in Millipore water for 24 h at room temperature (RT) to achieve a 0.1 M solution of BDA, which was immediately used for synthesis.

##### *cis*-2-[^13^C_4_]-Butene-1,4-dial

*cis*-2-[^13^C_4_]-Butene-1,4-dial ([^13^C_4_]-BDA) was prepared by hydrolysis of 0.1 M 2,5-diacetoxy-2,5-[^13^C_4_]-dihydrofuran in Millipore water for 24 h at RT to achieve a 0.1 M solution of [^13^C_4_]-BDA, which was immediately used for synthesis.

#### *Synthesis of [*^*12*^*C]-furan-dependent metabolites*

##### GSH-BDA

Glutathione (273 µmol in 2.729 mL 0.1 M potassium phosphate buffer, pH 7.4) was added to 2.729 mL BDA (0.1 M, 273 µmol). The solution was brought to a final volume of 6 mL with 0.1 M potassium phosphate buffer and incubated at 37 °C for 24 h with constant shaking. The product was purified by semipreparative HPLC (ZORBAX Eclipse XDB C18, Agilent, 5 µm, 9.4 × 250 mm, 80 Å) with 0.1% formic acid in water (solvent A) and 0.1% formic acid in acetonitrile (solvent B) at a flow rate of 4 mL/min using HPLC method 1 (Table 1). After lyophilization the yield of the purified product was 17.9 mg (18.5%). The structure was confirmed by LC–MS/MS (Supplementary Figure S1). ESI–MS/MS (pos): 356 [M^+1^], 338 [M^+1^–H_2_O], 253 [M^+1^–H_2_O–CO–CH_2_–NH–CO], 210 [M^+1^–CO–NH–CH_2_–COOH–CO_2_], 136 [M^+1^–CO–NH–CH_2_–COOH–COOH–C_2_H_6_–NH–CO].

**Table 1:** Semipreparative HPLC methods for purification of synthesized reference substances and internal standards

Method	Compound	Gradient	Detection (nm)
1	GSH-BDA	5 min gradient from 95% A and 5% B to 80% A and 20% B; 18 min gradient to 75% A and 25% B; 1 min gradient to 95% and 5% B (total: 24 min)	238
2	NAcLys-BDA NAcLys-[^13^C_4_]-BDA	12 min gradient from 95% A and 5% B to 70% A and 30% B; 3 min gradient to 95% and 5% B (total: 15 min)	238
3	NAcCys-BDA-NAcLys NAcCys-[^13^C_4_]-BDA-NAcLys [^13^C_2_]-NAcCys-BDA-NAcLys	1 min gradient from 95% A and 5% B to 77% A and 23% B; 13 min at 77% A and 23% B; 1 min gradient to 95% A and 5% B (total: 15 min)	254
4	NAcCys-BDA-NAcLys-SO NAcCys-[^13^C_4_]-BDA-NAcLys-SO [^13^C_2_]-NAcCys-BDA-NAcLys-SO	7 min gradient from 95% A and 5% B to 65% A and 35% B; 6 min gradient to 40% A and 60% B; 2 min gradient to 95% A and 5% B (total: 15 min)	254
5	[^13^C_6_^15^N_2_]-Lys-BDA	1 min gradient from 95% A and 5% B to 90% A and 10% B; 9 min at 90% A and 10% B; 2 min gradient to 70% A and 30% B; 3 min gradient to 95% A and 5% B (total: 15 min)	238
6	NAc-[^13^C_6_^15^N_2_]-Lys-BDA	10 min gradient from 95% A and 5% B to 90% A and 10% B; 1 min gradient to 75% A and 25% B; 4 min gradient to 95% A and 5% B (total: 15 min)	238

##### NAcLys-BDA

*N*-Acetyl-lysine (379 µmol in 1.01 mL 0.1 M potassium phosphate buffer, pH 7.4) was added to 3.79 mL BDA (0.1 M, 379 µmol). The solution was brought to a final volume of 6 mL with 0.1 M potassium phosphate buffer and incubated at 37 °C for 24 h with constant shaking. The product was purified by semipreparative HPLC (ZORBAX Eclipse XDB C18, Agilent, 5 µm, 9.4 × 250 mm, 80 Å) with 0.1% formic acid in water (solvent A) and 0.1% formic acid in acetonitrile (solvent B) at a flow rate of 4 mL/min using HPLC method 2 (Table 1). After lyophilization 13.9 mg (14.4%) of the purified product were obtained. The structure was confirmed by LC–MS/MS (Supplementary Figure S2). ESI–MS/MS (pos): 255 [M^+1^], 237 [M^+1^–H_2_O], 213 [M^+1^–CO–CH_2_], 209 [M^+1^–H_2_O–CO], 167 [M^+1^–H_2_O–CO–C_2_H_2_O], 150 [M^+1^–H_2_O–CO–CO–CH_3_–NH_2_].

##### NAcCys-BDA-NAcLys

*N*-Acetyl-l-cysteine (337 µmol in 1.685 mL 0.1 M potassium phosphate buffer, pH 7.4) was added to 3.37 mL BDA (0.1 M, 337 µmol). The solution was incubated for 15 min at 37 °C followed by the addition of 1.685 mL *N*-Acetyl-lysine (337 µmol in 0.1 M potassium phosphate buffer). The reaction mixture was brought to a final volume of 8 mL with 0.1 M potassium phosphate buffer and incubated for 24 h at 37 °C with constant shaking. The product was purified by semipreparative HPLC (ZORBAX Eclipse XDB C18, Agilent, 5 µm, 9.4 × 250 mm, 80 Å) with 0.1% formic acid in water (solvent A) and 0.1% formic acid in acetonitrile (solvent B) at a flow rate of 4 mL/min using HPLC method 3 (Table 1). After lyophilization the yield of the purified product was 49.8 mg (37.2%). The structure was confirmed by LC–MS/MS (Supplementary Figure S3). ESI–MS/MS (pos): 400 [M^+1^], 382 [M^+1^–H_2_O], 364 [M^+1^–H_2_O–H_2_O], 295 [M^+1^–H_2_O–CO–NH_2_–CO–CH_3_], 271 [M^+1^–C_2_H_2_–NH–CO–CH_3_–COOH], 166 [M^+1^–C_2_H_3_–NH–CO–CH_3_–COOH–H_2_O–CO–NH–CO–CH_3_].

##### NAcCys-BDA-NAcLys sulfoxide

Meta-chloroperbenzoic acid (26 µmol in 0.35 mL methanol) was added to a solution of purified NAcCys-BDA-NAcLys (26 µmol) in 2 mL methanol and dichloromethane (1:1) at –78 °C (total volume: 2.35 mL). The reaction mixture was stirred at –78 °C for 1 h and was then incubated for another 1 h at −78 °C without stirring, before warming up to RT. The product was immediately purified by semipreparative HPLC (ZORBAX Eclipse XDB C18, Agilent, 5 µm, 9.4 × 250 mm, 80 Å) with 0.1% formic acid in water (solvent A) and 0.1% formic acid in acetonitrile (solvent B) at a flow rate of 4 mL/min using HPLC method 4 (Table 1). After lyophilization the product yield was 5.8 mg (53.2%). The structure was confirmed by LC–MS/MS (Supplementary Figure S4). ESI–MS/MS (pos): 416 [M^+1^], 321 [M^+1^–H_2_O–NH_2_–CO–CH_3_–H_2_O], 269 [M^+1^–C_2_H_4_–COOH–NH–CH_3_–CO_2_], 239 [M^+1^–SO–C_2_H_2_–COOH–NH–CO–CH_3_], 223 [M^+1^–SO–C_2_H_2_–COOH–NH–CO–CH_2_–OH], 182 [M^+1^–SO–C_2_H_2_–COOH–NH–CO–CH_2_–NH–CO–CH_3_].

#### *Synthesis of [*^*13*^*C*_*4*_*]-furan-dependent metabolites*

##### GSH-[^13^C_4_]-BDA

Glutathione (419 µmol in 2.60 mL 0.1 M potassium phosphate buffer, pH 7.4) was added to 4.19 mL [^13^C_4_]-BDA (0.1 M, 419 µmol). The solution was brought to a final volume of 7 mL with 0.1 M potassium phosphate buffer and incubated at 37 °C for 24 h with constant shaking. The product was purified by semipreparative HPLC (ZORBAX Eclipse XDB C18, Agilent, 5 µm, 9.4 × 250 mm, 80 Å) with 0.1% formic acid in water (solvent A) and 0.1% formic acid in acetonitrile (solvent B) at a flow rate of 4 mL/min using HPLC method 1 (Table 1). Synthesis of GSH-[^13^C_4_]-BDA did not yield weighable quantities. The structure of the product was confirmed by LC–MS/MS (Supplementary Figure S1). ESI–MS/MS (pos): 360 [M^+1^], 342 [M^+1^–H_2_O], 257 [M^+1^–H_2_O–CO–CH_2_–NH–CO], 214 [M^+1^–CO–NH–CH_2_–COOH–CO_2_], 140 [M^+1^–CO–NH–CH_2_–COOH–COOH–C_2_H_6_–NH–CO].

##### NAcLys-[^13^C_4_]-BDA

*N*-Acetyl-l-lysine (417 µmol in 1.60 mL 0.1 M potassium phosphate buffer, pH 7.4) was added to 4.17 mL [^13^C_4_]-BDA (0.1 M, 417 µmol). The solution was brought to a final volume of 6 mL with 0.1 M potassium phosphate buffer and incubated at 37 °C for 24 h with constant shaking. The product was purified by semipreparative HPLC (ZORBAX Eclipse XDB C18, Agilent, 5 µm, 9.4 × 250 mm, 80 Å) with 0.1% formic acid in water (solvent A) and 0.1% formic acid in acetonitrile (solvent B) at a flow rate of 4 mL/min using HPLC method 2 (Table 1). After lyophilization the purified product was obtained in yield of 10.5 mg (9.8%). The structure was confirmed by LC–MS/MS (Supplementary Figure S2). ESI–MS/MS (pos): 259 [M^+1^], 241 [M^+1^–H_2_O], 217 [M^+1^–CO–CH_2_], 213 [M^+1^–H_2_O–CO], 171 [M^+1^–H_2_O–CO–C_2_H_2_O], 154 [M^+1^–H_2_O–CO–CO–CH_3_–NH_2_].

##### NAcCys-[^13^C_4_]-BDA-NAcLys

*N*-Acetyl-cysteine (359 µmol in 1.18 mL 0.1 M potassium phosphate buffer, pH 7.4) was added to 3.59 mL [^13^C_4_]-BDA (0.1 M, 359 µmol). The solution was incubated for 15 min at 37 °C followed by the addition of 1.4 mL *N*-Acetyl-lysine (359 µmol in 0.1 M potassium phosphate buffer) to a final volume of 6.17 mL and an incubation for 24 h at 37 °C with constant shaking. The product was purified by semipreparative HPLC (ZORBAX Eclipse XDB C18, Agilent, 5 µm, 9.4 × 250 mm, 80 Å) with 0.1% formic acid in water (solvent A) and 0.1% formic acid in acetonitrile (solvent B) at a flow rate of 4 mL/min using HPLC method 3 (Table 1). Lyophilization yielded 43.1 mg (29.8%). The structure was confirmed by LC–MS/MS (Supplementary Figure S3). ESI–MS/MS (pos): 404 [M^+1^], 386 [M^+1^–H_2_O], 368 [M^+1^–H_2_O–H_2_O], 299 [M^+1^–H_2_O–CO–NH_2_–CO–CH_3_], 275 [M^+1^–C_2_H_2_–NH–CO–CH_3_–COOH], 170 [M^+1^–C_2_H_3_–NH–CO–CH_3_–COOH–H_2_O–CO–NH–CO–CH_3_].

##### NAcCys-[^13^C_4_]-BDA-NAcLys sulfoxide

Meta-chloroperbenzoic acid (59 µmol in 1.015 mL methanol) was added to a solution of purified NAcCys-[^13^C_4_]-BDA-NAcLys (59 µmol) in 5 mL methanol and dichloromethane (1:1) at −78 °C and stirred for 1 h (total volume: 6.015 mL). Before warming up to RT, the reaction mixture was cooled for another hour without stirring. The product was immediately purified by semipreparative HPLC (ZORBAX Eclipse XDB C18, Agilent, 5 µm, 9.4 × 250 mm, 80 Å) with 0.1% formic acid in water (solvent A) and 0.1% formic acid in acetonitrile (solvent B) at a flow rate of 4 mL/min using HPLC method 4 (Table 1). Lyophilization yielded 14.8 mg (59.7%). The structure was confirmed by LC–MS/MS (Supplementary Figure S4). ESI–MS/MS (pos): 420 [M^+1^], 325 [M^+1^–H_2_O–NH_2_–CO–CH_3_–H_2_O], 273 [M^+1^–C_2_H_4_–COOH–NH–CH_3_–CO_2_], 243 [M^+1^–SO–C_2_H_2_–COOH–NH–CO–CH_3_], 227 [M^+1^–SO–C_2_H_2_–COOH–NH–CO–CH_2_–OH], 186 [M^+1^–SO–C_2_H_2_–COOH–NH–CO–CH_2_–NH–CO–CH_3_].

#### Synthesis of internal standards

##### NAc-[^13^C_6_^15^N_2_]-Lys-BDA

l-[^13^C_6_^15^N_2_]-lysine (145 µmol in 2 mL 0.1 M potassium phosphate buffer, pH 7.4) was added to 1.45 mL BDA (0.1 M, 145 µmol). The solution was brought to a final volume of 6 mL with 0.1 M potassium phosphate buffer and incubated at 37 °C for 24 h with constant shaking. The adduct [^13^C_6_^15^N_2_]-Lys-BDA was purified by semipreparative HPLC (ZORBAX Eclipse XDB C18, Agilent, 5 µm, 9.4 × 250 mm, 80 Å) with 0.1% formic acid in water (solvent A) and 0.1% formic acid in acetonitrile (solvent B) at a flow rate of 4 mL/min using HPLC method 5 (Table 1). To a solution of purified [^13^C_6_^15^N_2_]-Lys-BDA (31 µmol in 3 mL H_2_O), acetic anhydride (37 µmol) was added in portions of 1 µL at 2–5 °C. After each addition of acetic anhydride, the reaction mixture was neutralized with 0.25 M NaOH. At the end of the synthesis, 0.1 M formic acid was added to achieve pH 7.0. The product was purified by semipreparative HPLC (ZORBAX Eclipse XDB C18, Agilent, 5 µm, 9.4 × 250 mm, 80 Å) with 0.1% formic acid in water (solvent A) and 0.1% formic acid in acetonitrile (solvent B) at a flow rate of 4 mL/min using HPLC method 6 (Table 1). The structure was confirmed by LC–MS/MS (Supplementary Figure S2). ESI–MS/MS (pos): 263 [M^+1^], 245 [M^+1^–H_2_O], 221 [M^+1^–CO–CH_2_], 216 [M^+1^–H_2_O–^13^CO], 174 [M^+1^–H_2_O–^13^CO–CO–CH_2_], 156 [M^+1^–H_2_O–^13^CO–CO–CH_3_–^15^NH_2_].

##### [^13^C_2_]-NAcCys-BDA-NAcLys

[^13^C_2_]-*N*-Acetyl-l-cysteine (139 µmol in 1.00 mL 0.1 M potassium phosphate buffer, pH 7.4) was added to 1.39 mL BDA (0.1 M, 139 µmol). The solution was incubated for 15 min at 37 °C followed by the addition of 0.964 mL *N*-Acetyl-l-lysine (139 µmol in 0.1 M potassium phosphate buffer). The reaction mixture was brought to a final volume of 4.5 mL with 0.1 M potassium phosphate buffer and incubated for 24 h at 37 °C with constant shaking. The product was purified by semipreparative HPLC (ZORBAX Eclipse XDB C18, Agilent, 5 µm, 9.4 × 250 mm, 80 Å) with 0.1% formic acid in water (solvent A) and 0.1% formic acid in acetonitrile (solvent B) at a flow rate of 4 mL/min using HPLC method 3 (Table 1). Lyophilization yielded 29.7 mg (53.6%). The structure was confirmed by LC–MS/MS (Supplementary Figure S3). ESI–MS/MS (pos): 402 [M^+1^], 384 [M^+1^–H_2_O], 366 [M^+1^–H_2_O–H_2_O], 295 [M^+1^–H_2_O–^13^CO–NH_2_–^13^CO–CH_3_], 271 [M^+1^–C_2_H_2_–NH–^13^CO–CH_3_–^13^COOH], 166 [M^+1^–C_2_H_3_–NH–^13^CO–CH_3_–^13^COOH–H_2_O–CO–NH–CO–CH_3_].

##### [^13^C_2_]-NAcCys-BDA-NAcLys sulfoxide

Meta-chloroperbenzoic acid (39 µmol in 0.604 mL methanol) was added to a solution of purified [^13^C_2_]-NAcCys-BDA-NAcLys (39 µmol) in 3 mL methanol and dichloromethane (1:1) at −78 °C and stirred for 1 h (total volume: 3.604 mL). Before warming up to RT, the reaction mixture was cooled for another 1 h without stirring. The product was immediately purified by semipreparative HPLC (ZORBAX Eclipse XDB C18, Agilent, 5 µm, 9.4 × 250 mm, 80 Å) with 0.1% formic acid in water (solvent A) and 0.1% formic acid in acetonitrile (solvent B) at a flow rate of 4 mL/min using HPLC method 4 (Table 1). Lyophilization yielded 9.8 mg (60.2%). The structure was confirmed by LC–MS/MS (Supplementary Figure S4). ESI–MS/MS (pos): 418 [M^+1^], 322 [M^+1^–H_2_O–NH_2_–^13^CO–CH_3_–H_2_O], 269 [M^+1^–C_2_H_4_–^13^COOH–NH–CH_3_–^13^CO_2_], 239 [M^+1^–SO–C_2_H_2_-^13^COOH–NH–^13^CO–CH_3_], 223 [M^+1^–SO–C_2_H_2_–^13^COOH–NH–^13^CO–CH_2_–OH], 182 [M^+1^–SO–C_2_H_2_–^13^COOH–NH–^13^CO–CH_2_–NH–CO–CH_3_].

### Animal experiments

Animal experiments were performed according to national animal welfare regulations after authorization by the local authorities (Regierung von Unterfranken, AZ RUF-55.2.2-2532-2-1256-20). A total of sixty 6–7 weeks old male and female F344/DuCrl rats (Charles River, Sulzfeld, Germany) were housed in groups of five in Macrolon cages with free access to sterilized (25 kGy) pelleted standard rat maintenance diet (SSNIFF, Soest, Germany) and tap water to acclimatize for 12 days prior to treatment. Room temperature was maintained at 22 ± 2 °C with a relative humidity of 55 ± 10% and a day/night cycle of 14/10 h. Rats were transferred into individual metabolic cages 24 h prior to treatment with free access to sterilized (25 kGy) ground standard rat maintenance and tap water ad libitum. Rats (*n* = 5 per dose and sex, bw males: 142–180 g; bw females: 110–129 g) received a single dose of [^13^C_4_]-furan dissolved in corn oil (4 mL/kg bw) at doses of 0, 0.1, 1, 10, 100 and 1000 µg/kg bw by oral gavage.

Dosing solutions were freshly prepared immediately prior to administration. The entire content of a glass ampulla containing 10 mg (nominal amount) [^13^C_4_]-furan (cooled to −80 °C to prevent evaporation loss) was first diluted in 10 mL corn oil. This solution was further diluted with corn oil to prepare stock solutions containing 50 and 450 µg/mL [^13^C_4_]-furan. These stock solutions served to prepare 0.25 and 0.025 µg/mL dosing solutions for the 0.1 and 1 µg/kg bw dose groups, and 2.5, 25 and 250 µg/mL dosing solutions for the 10, 100 and 1000 µg/kg bw dose groups, respectively. Considering the volatility of furan, an aliquot of each stock solution (50 and 450 µg/mL) was retained for subsequent GC–MS analysis to confirm the [^13^C_4_]-furan content (2.6. HS–GC–MS analysis of [^13^C_4_]-furan stock solutions).

Urine samples were collected on ice for 24 h prior and for 6 days after treatment. During the first 24 h after treatment, urine was collected every 8 h, followed by collection intervals of 24 h until sacrifice. Urine volume was recorded at the end of the collection period and aliquots were stored at −80 °C until further analysis. To reduce furan intake via animal feed during acclimatization and throughout the study, sterilized (25 kGy) pelleted and ground feed was used instead of autoclaved feed. The furan content in animal feed was determined by HS–GC–MS as described below (2.7).

### Sample preparation

Urine aliquots were thawed at 4–8 °C and vortexed. Urine was diluted with water (LC–MS/MS grade) at dilution factors of 2–200 to achieve analyte concentrations within the linear range of the calibration curves. To a volume of 90 µL diluted urine, 10 µL of internal standard mix and 1 µL of 8 M HCl were added. The internal standard mix contained isotopically labelled [^13^C_2_^15^N_1_]-GSH-BDA (1.44 µM), [^13^C_6_^15^N_2_]-NAcLys-BDA (0.92 µM), [^13^C_2_]-NAcCys-BDA-NAcLys (0.31 µM) and [^13^C_2_]-NAcCys-BDA-NAcLys sulfoxide (0.35 µM). The solutions were centrifuged at 4 °C and 15,000 g for 10 min.

### LC–MS/MS analyses

LC–MS/MS analyses were performed with a Triple Quad 5500+ QTRAP mass spectrometer (Applied Biosystems/MDS Sciex, Darmstadt, Germany) coupled to an Agilent 1100 HPLC and Agilent 1100 autosampler (Agilent, Waldbronn, Germany). Analytes (10 µL per sample) were separated on a Synergi Polar-RP analytical column (4 µm, 150 × 2 mm, 80 Å, Phenomenex Inc.) with water (containing 0.1% (v/v) formic acid) as solvent A and methanol (containing 0.1% (v/v) formic acid) as solvent B at a flow rate of 0.3 mL/min. Solvent A was held at 100% for 3 min, followed by a linear gradient to 20% A/80% B in 7 min. These conditions were held for 2 min before decreasing to 10% A/90% B. After 2 min at 90% B, the gradient was returned to initial conditions of 100% A/0% B within 2 min and remained until the end of the run (22 min).

Mass spectrometric analysis was performed using electrospray ionization operating in positive ion mode and multiple reaction monitoring (MRM) mode with an ion spray voltage of 5500 V and a source temperature of 500 °C. Nitrogen was used as ion spray (50 psi), drying gas (60 psi), curtain gas (40 psi) and collision gas. Compound specific ESI–MS/MS-parameters are given in Table 2. Data were recorded by Analyst 1.7.3 software (Applied Biosystems/MDS Sciex, Darmstadt, Germany).

**Table 2:** Compound-specific LC–ESI–MS/MS parameters for [^12^C]-furan- and [^13^C_4_]-furan-dependent metabolites and internal standards. Shown are precursor ion, product ion (quantifier (Qn) and qualifier transition (Ql)), declustering potential (DP), entrance potential, cell entrance potential (CEP), collision energy (CE) and cell exit potential (CXP)

Compound	RT (min)	Precursor ion (m/z)	Product ion (m/z)	DP (V)	EP (V)	CE (V)	CXP (V)
GSH-BDA	11.31	356.0	210.0 (Qn)	106	10	35	12
			136.0 (Ql)		10	65	16
GSH-[^13^C_4_]-BDA		360.1	214.0 (Qn)	101	10	33	14
			140.0 (Ql)		10	65	22
[^13^C_2_^15^N_1_]-GSH-BDA		359.2	210.2 (Qn)	91	10	33	14
			341.0 (Ql)		10	33	14
NAcLys-BDA	11.47	255.0	209.2 (Qn)	71	10	19	14
			167.2 (Ql)		10	25	20
NAcLys-[^13^C_4_]-BDA		259.1	213.1 (Qn)	86	10	19	14
			171.2 (Ql)		10	25	20
NAc-[^13^C_6_^15^N_2_]-Lys-BDA		263.0	216.2 (Qn)	81	10	19	14
			174.1 (Ql)		10	25	20
NAcCys-BDA-NAcLys	13.37	400.1	271.1 (Qn)	96	10	23	16
			166.0 (Ql)		10	39	20
NAcCys-[^13^C_4_]-BDA-NAcLys		404.1	275.1 (Qn)	101	10	23	18
			170.0 (Ql)		10	41	20
[^13^C_2_]-NAcCys-BDA-NAcLys		402.1	271.1 (Qn)	106	10	41	22
			166.0 (Ql)		10	35	16
NAcCys-BDA-NAcLys-SO	11.68	416.1	269.0 (Qn)	106	10	19	16
			129.9 (Ql)		10	35	16
NAcCys-[^13^C_4_]-BDA-NAcLys-SO		420.1	273.1 (Qn)	106	10	19	16
			130.0 (Ql)		10	35	14
[^13^C_2_]-NAcCys-BDA-NAcLys-SO		418.1	269.2 (Qn)	106	10	19	16
			132.0 (Ql)		10	35	16

Seven-point calibration curves were prepared by spiking water with appropriate volumes of working standard solution, containing 100 ng/mL of each metabolite. Calibration was linear in the range of 1.25–80 ng/mL. Working standard solution was prepared from individual stock solutions (1 mg/mL) of the respective metabolite in water. Due to the low yield of GSH-[^13^C_4_]-BDA and limited availability/significant costs of 2,5-diacetoxy-2,5-[^13^C_4_]-dihydrofuran required for synthesis, quantitation of GSH-[^13^C_4_]-BDA was performed using GSH-BDA. To 90 µL of each calibration standard 10 µL of internal standard mix and 1 µL of 8 M HCl were added. Solutions were then vortexed and centrifuged at 4 °C and 15,000 g for 10 min. The peak area ratios (analyte peak area/internal standard peak area) were used for quantitation. To ensure method precision during the analytical run, quality controls of 20 ng/mL were measured after every tenth sample. Limits of detection (LOD) and quantitation (LOQ) for each reference substance defined as a signal-to-noise ratio of 1:3 and 1:7, respectively, are provided in Table 3.

**Table 3:** Limits of detection (LOD) and quantitation (LOQ) of [^12^C]- and [^13^C_4_]-labeled reference substances for the quantitative LC–ESI–MS/MS method used for urine sample analysis

	LOD [ng/mL]	LOQ [ng/mL]
GSH-BDA	0.29 (0.81 nM)	0.67 (1.90 nM)
NAcLys-BDA	0.27 (1.06 nM)	0.63 (2.47 nM)
NAcCys-BDA-NAcLys	0.50 (1.26 nM)	1.17 (2.94 nM)
NAcCys-BDA-NAcLys sulfoxide	0.47 (1.12 nM)	1.09 (2.62 nM)
NAcLys-[^13^C_4_]-BDA	0.28 (1.08 nM)	0.65 (2.53 nM)
NAcCys-[^13^C_4_]-BDA-NAcLys	0.32 (0.80 nM)	0.76 (1.87 nM)
NAcCys-[^13^C_4_]-BDA-NAcLys sulfoxide	0.48 (1.15 nM)	1.12 (2.68 nM)

### ***HS-GC–MS analysis of [***^***13***^***C***_***4***_***]-furan in stock solutions***

The [^13^C_4_]-furan content of the stock solutions used to prepare the dosing solutions was analyzed by headspace GC–MS analysis following the FDA standard addition method for determination of furan in foods with minor modifications (Food and Drug Administration 2004). Briefly, GC–MS analysis was carried out using an Agilent 6890 GC coupled to an Agilent 5973 MSD (Hewlett-Packard). Chromatographic separation was performed on a HP-Plot Q capillary column (30 m × 0.32 mm, 20 µm phase thickness) with helium as carrier gas at a constant flow rate of 1.0 mL/min. The gas chromatograph operated with a split ratio of 10:1 and an injector temperature maintained at 200 °C. The gas chromatographic oven program started with an initial temperature of 50 °C for 1 min, an increase to 260 °C at a rate of 20 °C/min. The temperature was then held at 260 °C for 2.5 min. The total chromatographic run time was 14 min. The mass spectrometer was operated in electron-ionization mode at 70 eV with a source temperature of 230 °C and a MS-quad temperature of 150 °C. Data were acquired in selected ion monitoring (SIM) mode. The following ions were selected as quantifier (Qn) and qualifier (Ql): [^13^C_4_]-furan, *m/z* 72 (Qn) and 42 (Ql); furan, *m/z* 68 (Qn) and 39 (Ql); acetone, *m/z* 43 (Qn) and 58 (Ql). Dwell time was set at 50 ms for each ion. The retention time of [^13^C_4_]-furan and furan was 8.45 min, while acetone used as internal standard eluted at 9.05 min. The concentration of [^13^C_4_]-furan in the stock solutions was determined using standard addition. To obtain standard addition curves, stock solutions containing a nominal concentration of 50 µg/mL [^13^C_4_]-furan in corn oil were spiked with 25, 50 and 100 µg furan using a working solution of 5 mg/mL furan in methanol. Stock solutions of 450 µg/mL [^13^C_4_]-furan in corn oil were spiked with 250, 500 and 1000 µg furan using a working solution of 50 mg/mL furan in methanol. Working solutions were freshly prepared before use. Samples were fortified with 20 µL of internal standard solution of acetone in methanol, using a 50 mg/mL internal standard solution for 450 µg/mL stock solutions and a 5 mg/mL internal standard solution for 50 µg/mL stock solutions. Following incubation for 10 min at 60 °C, a volume of 1 mL gas phase was injected into the GC–MS system. Quantitation was conducted by evaluating peak areas in TIC modus and by plotting the area ratio of analyte and internal standard against the amount of spiked furan.

### HS-GC–MS analyses of furan in animal feed

Furan was analyzed using a headspace-gas chromatography/mass spectrometry (HS–GC–MS) procedure, initially developed and validated for baby foods (Lachenmeier et al. 2009). Briefly, approximately 1 g of animal feed sample was cryo-milled and added to a headspace vial with 90 mL water and measured using HS–GC–MS. Deuterated furan (furan-d_4_) was used as internal standard, and the quantification was conducted using a multipoint calibration method. Analysis was conducted on a gas chromatograph coupled with mass spectrometer (Agilent 6890/5973) and a headspace sampler Combi PAL MXY 02-01B (Agilent, Waldbronn, Germany). Gas chromatography column: HP-Plot Q, 30 m, 0.32 mm I.D., film 20 µm (Agilent, Waldbronn, Germany). For further methodological details, see (Lachenmeier et al. 2009).

## Results

To discriminate between the externally applied furan dose and background exposure via feed and/or endogenous formation and to establish the correlation between external dose and biomarker concentration, urinary excretion of [^13^C_4_]-labeled and unlabeled furan-dependent metabolites was simultaneously monitored for 6 days after a single oral dose of [^13^C_4_]-furan. Based on this experimental design, [^13^C_4_]-furan-dependent metabolites serve to monitor exposure resulting from the administered external dose, while [^12^C]-furan-dependent metabolites may represent either endogenous formation or background exposure to furan e.g., via animal feed. Considering the volatility of furan and concerns for evaporation loss during preparation of the dosing solutions, the [^13^C_4_]-furan content of the stock solutions used to prepare the dosing solutions was analyzed by GC–MS analysis. These analyses revealed slightly higher concentrations than the nominal concentrations (Supplementary Table S1), presumably due to a slightly higher than nominal amount of [^13^C_4_]-furan supplied in the glass ampulla. Based on these results, the administered doses were corrected accordingly (nominal dose: 0.1, 1, 10, 100 1000 µg/kg bw; actual dose males: 0.1, 1, 14, 141, 1410 µg/kg bw, respectively, 1.4, 14, 139, 1389, 13,889 nmol/kg bw; actual dose females: 0.1, 1, 14, 143, 1430 µg/kg bw, respectively 1.6, 16, 199, 1988, 19,880 nmol/kg bw) (Supplementary Table S1). Excretion rates of [^13^C_4_]-furan metabolites were calculated based on the actual dose applied.

**Fig. 2:**
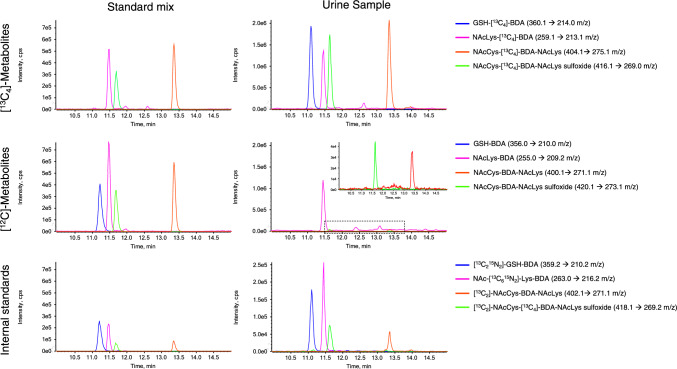
Extracted ion chromatograms of GSH-BDA (t_R_ 11.3 min), NAcLys-BDA (t_R_ 11.5 min), NAcCys-BDA-NAcLys (t_R_ 13.4 min) and NAcCys-BDA-NAcLys sulfoxide (11.7 min) as [^13^C_4_]-furan metabolites, [^12^C]-furan metabolites and matching internal standards obtained from a standard mix solution and a 16 h urine sample from a male F344/DuCrl rat treated with 1000 µg/kg bw [^13^C_4_]-furan

For accurate quantification of urinary furan metabolites via stable isotope dilution ESI–LC–MS/MS, unlabeled and [^13^C_4_]-labeled reference compounds as well as isotopically labeled internal standards of the putative biomarkers GSH-BDA, NAcLys-BDA, NAcCys-BDA-NAcLys and NAcCys-BDA-NAcLys sulfoxide were synthesized and purified by preparative HPLC. Identity of the purified reference compounds was confirmed by mass spectrometry (Supplementary Figures S1–S4). A previous LC–MS/MS method (Karlstetter and Mally 2020) was adapted to allow separation and quantitative analysis of the four [^13^C_4_]-labeled and unlabeled furan-dependent metabolites by isotope dilution LC–ESI–MS/MS (Fig. 2). LC–MS/MS analysis confirmed the presence of GSH-[^13^C_4_]-BDA, NAcLys-[^13^C_4_]-BDA, NAcCys-[^13^C_4_]-BDA-NAcLys and NAcCys-[^13^C_4_]-BDA-NAcLys sulfoxide in urine of male and female F344/DuCrl rats treated with [^13^C_4_]-furan at 1, 10, 100 and 100 µg/kg bw (Fig. 2). [^13^C_4_]-labeled metabolites were below the LODs in urine of rats treated with [^13^C_4_]-furan at 0.1 µg/kg bw, while NAcCys-[^13^C_4_]-BDA-NAcLys sulfoxide was also not detectable in urine of female rats administered [^13^C_4_]-furan at 1 µg/kg bw. Due to an unidentified interfering peak with same mass transitions and similar retention time, possibly due to the high background of NAcLys-BDA and the natural abundance of the ^13^C isoptope, NAcLys-[^13^C_4_]-BDA was not quantifiable in urine of male rats at dose levels below 100 µg/kg bw and in urine of female rats at dose levels below 1000 µg/kg bw.

**Fig. 3:**
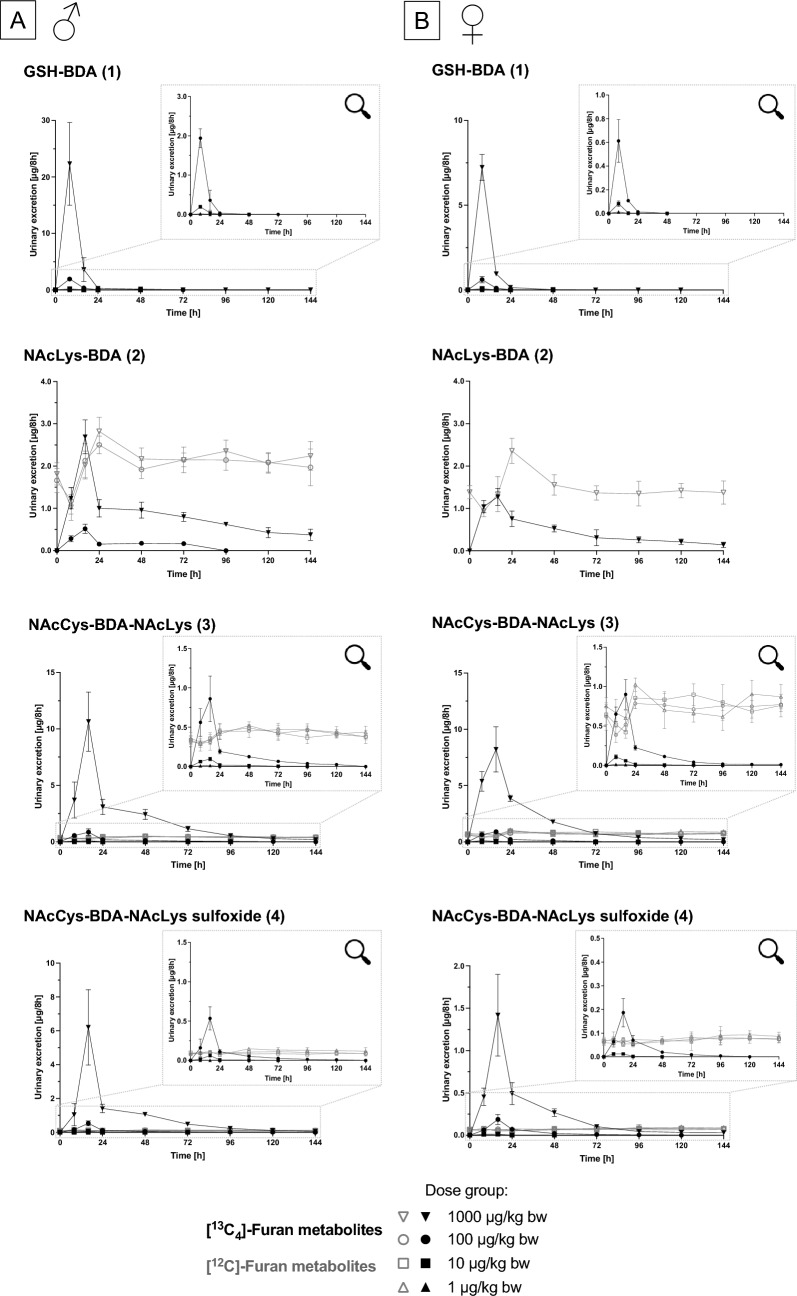
Urinary excretion of GSH-BDA, NAcLys-BDA, NAcCys-BDA-NAcLys and NAcCys-BDA-NAcLys sulfoxide as [^13^C_4_]-furan-derived metabolites (filled symbols) and corresponding unlabeled compounds (open symbols) in urine of male (**A**) and female (**B**) F344/DuCrl rats. Inserts present zoomed plots of the highlighted areas of the diagrams, showing metabolite excretion in the lower dose groups (100–1 µg/kg bw [^13^C_4_]-furan). Data are presented as mean ± standard deviation (*n* = 5) of the amount of metabolite excreted within 8 h

**Fig. 4:**
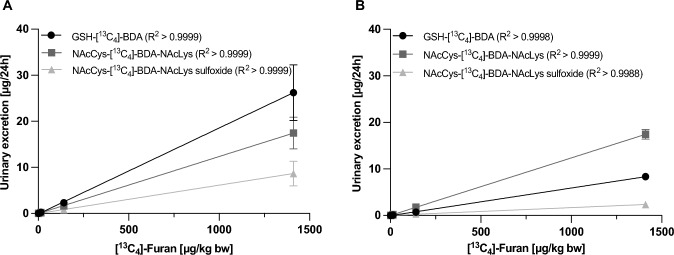
Linear correlation between external [^13^C_4_]-furan dose and 24 h urinary excretion of furan metabolites in male (**A**) and female (**B**) rats. Data are presented as mean ± standard deviation (*n* = 5)

GSH-[^13^C_4_]-BDA was rapidly excreted within the first 24 h after application of [^13^C_4_]-furan and was not detected at later sampling time points, whereas NAcLys-[^13^C_4_]-BDA, NAcCys-[^13^C_4_]-BDA-NAcLys and NAcCys-[^13^C_4_]-BDA-NAcLys sulfoxide were excreted in a slightly delayed manner (Fig. 3). Relative excretion rates of [^13^C_4_]-furan-derived metabolites were low. On average across dose-groups, around 5.0 and 4.1% of the administered dose were recovered in urine of male and female rats, respectively, within 24 h. In addition to the slightly lower recovery in females, gender differences were also evident in the metabolic profiles (Table 4), with GSH-[^13^C_4_]-BDA as the major metabolite in males and NAcCys-[^13^C_4_]-BDA-NAcLys as the most abundant metabolite in females. Excretion of GSH-[^13^C_4_]-BDA, NAcLys-[^13^C_4_]-BDA, NAcCys-[^13^C_4_]-BDA-NAcLys and NAcCys-[^13^C_4_]-BDA-NAcLys sulfoxide within 24 h accounted for 1.66–3.08%, 0.59%, 1.00–2.86% and 0.40–1.09% of the external [^13^C_4_]-furan dose in male rats, and 0.70–2.97%, 0.52%, 1.57–3.40% and 0.12–0.42% in females (Table 4). Across the administered dose range, a linear correlation between the external [^13^C_4_]-furan dose and urinary excretion of GSH-[^13^C_4_]-BDA, NAcCys-[^13^C_4_]-BDA-NAcLys and NAcCys-[^13^C_4_]-BDA-NAcLys sulfoxide was established in both male and female rats (Fig. 4). Although a dose-related increase in urinary NAcLys-[^13^C_4_]-BDA excretion was observed, correlation analysis was not performed for NAcLys-[^13^C_4_]-BDA, as accurate analysis of this metabolite was only possible in the two highest dose groups due to an interfering peak.

**Table 4:** Urinary 24 h excretion of GSH-[^13^C_4_]-BDA, NAcLys-[^13^C_4_]-BDA, NAcCys-[^13^C_4_]-BDA-NAcLys, NAcCys-[^13^C_4_]-BDA-NAcLys sulfoxide expressed as µg/24 h, nmol/24 h and fraction of the administered dose (%)^a^ after treatment of male and female F344/DuCrl rats with a single dose of [^13^C_4_]-furan, as well as 24 h background excretion of NAcLys-BDA, NAcCys-BDA-NAcLys and NAcCys-BDA-NAcLys sulfoxide

Metabolite	Sex	Excretion in		Furan dose (µg/kg bw)					
			Nominal	0	0.1	1	10	100	1000
			Actual^b^	0	0.1	1	14	140	1400
*[*^*13*^*C*_*4*_*]-Furan dependent metabolites*									
GSH-[^13^C_4_]-BDA	Male	µg/24 h		–	< 0.004	0.021 ± 0.005	0.23 ± 0.02	2.33 ± 0.22	26.2 ± 6.1
		nmol/24 h		–	< 0.011	0.058 ± 0.013	0.65 ± 0.06	6.50 ± 0.62	73.0 ± 16.9
		(%)		–	–	2.42	2.07	2.01	2.25
	Female	µg/24 h		–	< 0.005	0.012 ± 0.004	0.09 ± 0.02	0.73 ± 0.18	8.3 ± 0.7
		nmol/24 h		–	< 0.013	0.034 ± 0.012	0.24 ± 0.07	2.04 ± 0.51	23.2 ± 2.0
		(%)		–	–	1.84	0.99	0.88	1.01
NAcLys-[^13^C_4_]-BDA	Male	µg/24 h		–	–	–	–	0.96 ± 0.04	4.8 ± 0.3
		Nmol/24 h		–	–	–	–	3.71 ± 0.16	18.8 ± 1.1
		(%)		–	–	–	–	1.15	0.58
	Female	µg/24 h		–	–	–	–	–	3.1 ± 0.3
		nmol/24 h		–	–	–	–	–	11.9 ± 1.2
		(%)		–	–	–	–	–	0.52
NAcCys-[^13^C_4_]-BDA-NAcLys	Male	µg/24 h		–	< 0.005	0.025 ± 0.002	0.18 ± 0.01	1.61 ± 0.25	17.4 ± 3.5
		nmol/24 h		–	< 0.011	0.061 ± 0.005	0.44 ± 0.03	4.00 ± 0.62	43.3 ± 8.6
		(%)		–	-	2.54	1.41	1.24	1.33
	Female	µg/24 h		–	< 0.005	0.025 ± 0.002	0.18 ± 0.02	1.78 ± 0.18	17.5 ± 1.1
		nmol/24 h		–	< 0.013	0.061 ± 0.006	0.45 ± 0.05	4.41 ± 0.44	43.4 ± 2.7
		(%)		–	-	2.93	1.85	1.91	1.88
NAcCys-[^13^C_4_]-BDA-NAcLys sulfoxide	Male	µg/24 h		–	< 0.007	0.009 ± 0.002	0.09 ± 0.01	0.81 ± 0.13	8.7 ± 2.7
		nmol/24 h		–	< 0.016	0.022 ± 0.003	0.21 ± 0.03	1.93 ± 0.32	20.7 ± 6.4
		(%)		–	–	0.92	0.66	0.60	0.64
	Female	µg/24 h		–	< 0.008	< 0.009	0.03 ± 0.01	0.32 ± 0.06	2.4 ± 0.5
		nmol/24 h		–	< 0.018	< 0.021	0.06 ± 0.02	0.77 ± 0.15	5.6 ± 1.1
		(%)		–	–	–	0.26	0.33	0.24
*[*^*12*^*C]-Furan dependent metabolites (*= *background)*									
NAcLys-BDA	Male	µg/24 h		4.44 ± 1.09	5.50 ± 0.45	5.17 ± 0.51	5.51 ± 0.70	5.80 ± 0.31	5.87 ± 0.92
		nmol/24 h		17.5 ± 4.29	21.7 ± 1.78	20.3 ± 2.01	21.7 ± 2.76	22.8 ± 1.21	23.1 ± 3.63
	Female	µg/24 h		4.53 ± 0.24	4.39 ± 0.44	4.76 ± 0.33	4.64 ± 0.79	4.66 ± 0.49	4.64 ± 0.31
		nmol/24 h		17.8 ± 0.94	17.3 ± 1.74	18.7 ± 1.29	18.3 ± 3.10	18.3 ± 1.94	18.3 ± 1.22
NAcCys-BDA-NAcLys	Male	µg/24 h		0.88 ± 0.21	1.12 ± 0.13	1.10 ± 0.03	1.09 ± 0.19	1.05 ± 0.26	0.83 ± 0.27
		nmol/24 h		2.21 ± 0.53	2.79 ± 0.32	2.75 ± 0.09	2.72 ± 0.49	2.62 ± 0.66	2.07 ± 0.67
	Female	µg/24 h		2.06 ± 0.27	1.86 ± 0.14	2.30 ± 0.20	1.80 ± 0.32	1.69 ± 0.17	2.21 ± 0.25
		nmol/24 h		5.17 ± 0.67	4.65 ± 0.36	5.75 ± 0.51	4.50 ± 0.81	4.24 ± 0.43	5.54 ± 0.62
NAcCys-BDA-NAcLys sulfoxide	Male	µg/24 h		0.26 ± 0.02	0.24 ± 0.04	0.29 ± 0.04	0.29 ± 0.05	0.28 ± 0.04	0.38 ± 0.14
		nmol/24 h		0.63 ± 0.04	0.57 ± 0.10	0.69 ± 0.09	0.70 ± 0.11	0.67 ± 0.10	0.91 ± 0.34
	Female	µg/24 h		0.15 ± 0.03	0.19 ± 0.03	0.20 ± 0.02	0.18 ± 0.05	0.18 ± 0.02	0.17 ± 0.03
		nmol/24 h		0.37 ± 0.07	0.46 ± 0.08	0.48 ± 0.06	0.44 ± 0.12	0.44 ± 0.04	0.40 ± 0.06

^a^Dose adjusted based on analysis of the [^13^C_4_]-furan concentration in the stock solutions

^b^Actual doses for the 10, 100 and 1000 µg/kg bw dose groups reported in the table were rounded to two significant digits (actual dose males: 14, 141, 1410 µg/kg bw; actual dose females: 14, 143, 1430 µg/kg bw)

Analysis of unlabeled furan-derived metabolites revealed substantial, fairly constant urinary background levels of unlabeled NAcLys-BDA, NAcCys-BDA-NAcLys and NAcCys-BDA-NAcLys sulfoxide throughout the course of the study (Fig. 3). In male rats, urinary background excretion of NAcCys-BDA-NAcLys and NAcCys-BDA-NAcLys sulfoxide exceeded the concentration of the corresponding [^13^C_4_]-furan-derived metabolites at [^13^C_4_]-furan doses below 100 µg/kg bw, while excretion of unlabeled NAcLys-BDA exceeded excretion of NAcLys-[^13^C_4_]-BDA even at the highest [^13^C_4_]-furan dose of 1000 µg/kg bw. Similar background levels were observed in female rats, with background levels of NAcCys-BDA-NAcLys exceeding the urinary concentration of NAcCys-[^13^C_4_]-BDA-NAcLys at all dose levels < 1000 µg/kg bw. Consistent with previous work (Karlstetter and Mally 2020), GSH-BDA was identified as the only furan metabolite without background occurrence, supporting its suitability as a biomarker of external furan exposure (Figs. 2 and 3).

Based on the 24 h-exretion rates established via analysis of [^13^C_4_]-furan-dependent metabolites (Table 5), excretion of unlabeled NAcCys-BDA-NAcLys and NAcCys-BDA-NAcLys sulfoxide was estimated to correspond to a furan dose of 46 and 70 µg/kg bw/d in males and 94 and 135 µg/kg bw/d in females. To understand if the presence of furan in animal feed may account for these background levels, the content of furan in 25 kGy sterilized animal feed used in the present study vs. standard autoclaved animal feed was assessed by HS–GC–MS. In contrast to autoclaved feed, which was found to contain furan at 35 µg/kg feed, furan was below the LOQ (< 5 µg/kg) in 25 kGy sterilized ground animal feed. Pelleted 25 kGy sterilized animal feed that was fed during acclimatization up to 24 h before the start of the study was found to contain furan at 7.5 µg/kg feed. Based on the LOQ of 5 µg furan/kg feed and feed consumption data (mean daily feed consumption: 16.5 ± 3.3 g/d), furan exposure of animals via feed was estimated at < 0.6 µg/kg bw per day. Estimated intake of furan via feed (0.6 µg/kg bw) was thus about two orders of magnitude lower than background exposure estimated based on biomarker excretion (Table 5). In particular, the high background levels of NAcLys-BDA, which by far exceeded the levels of the BDA derived lysine-cysteine crosslinks, were estimated to correspond to a theoretical furan dose of 1.6 mg/kg bw per day in males and 2.1 mg/kg bw per day in females.

**Table 5:** Probable daily furan intakes (PDIs) corresponding to background excretion of furan metabolites [µg/24 h] based on relative excretion rates of the respective [^13^C_4_]-furan derived metabolites

Metabolite	Sex	Background excretion [nmol/24 h ]	Relative 24 h-excretion rate [^13^C_4_]-metabolite [% of external dose]	PDI^d^ [µg/kg bw/d]
GSH-BDA	Male	–	2.18^a^	–
	Female	–	1.18^a^	–
NAcLys-BDA	Male	21.22	0.59^b^	1646
	Female	18.12	0.52^b^	2095
NAcCys-BDA-NAcLys	Male	2.52	1.58^a^	70
	Female	4.98	2.14^a^	135
NAcCys-BDA-NAcLys sulfoxide	Male	0.70	0.69^a^	46
	Female	0.43	0.28^c^	94

PDIs were calculated using the following relative 24 h-excretion rates:

^a^Mean relative 24 h excretion rate across dose groups 1–1000 µg/kg bw

^b^Relative 24 h excretion rate of highest dose group 1000 µg/kg bw

^c^Mean relative 24 h excretion rate across dose groups 10–1000 µg/kg bw

^d^PDI calculation: $${\text{Probable daily intake}}\ ({\upmu} {\text{g/kg}}\ {\text{bw/d}}) = \frac{{\left({{\text{Background excretion}}\ {\text{(mol/24}}\,{\text{h}}) \times \frac{{\left[{{\text{13C4}}}\right] -{\text{Furan dose}}\;{\text{(mol)}}}}{{{\text{Excretion}}\left[ {{\text{13C4}}} \right] -{\text{metabolite}}\ {\text{(mol/24}}\,{\text{h)}}}}} \right) \times {\text{Molecular weight Furan}}\ ({\upmu} {\text{g/mol)}}}}{{\text{Body weight (kg)}}}$$

## Discussion

The overall aim of the present study was to test the validity of a biomarker-based approach to assess exposure to furan via food. Administration of isotopically labelled [^13^C_4_]-furan to rats across a wide dose range that included doses relevant to human exposure (0.1–10 µg/kg bw) allowed us to precisely establish the correlation between external dose and concentration of furan metabolites in urine over time and to discriminate against endogenous formation and furan intake via feed.

Although a linear correlation was observed between [^13^C_4_]-furan dose and urinary excretion of [^13^C_4_]-furan-dependent metabolites, the high background levels of NAcLys-BDA, NAcCys-BDA-NAcLys and NAcCys-BDA-NAcLys sulfoxide render these metabolites unsuitable as biomarkers of dietary furan. In particular, the constant high urinary background levels of NAcLys-BDA in the range of NAcLys-[^13^C_4_]-BDA concentrations observed in response to the highest dose of [^13^C_4_]-furan (1 mg/kg bw) and estimated to correspond to a furan intake of 1.6 and 2.1 mg/kg bw in male and female rats, respectively, provide clear evidence for a significant but yet unexplored source of furan or its metabolites. While our analyses of furan in animal feed appear to exclude animal feed as a significant source of furan exposure under the experimental conditions of our study, we cannot rule out the possibility that amino acid adducts of BDA, such as Lys-BDA, may be present in feed. This may be supported by the observation that urinary concentrations of NAcLys-BDA first declined during the day-time collection period on day 1 (8 h) and then continuously increased at the two later urine collection periods (16 and 24 h), and, therefore, corresponded with the animals´ nocturnal activity and eating/drinking behaviour. Alternatively, there is some evidence to suggest that furan and 2-butene-1,4-dial may be formed endogenously. Suggested pathways for endogenous formation include lipid peroxidation and 5’-oxidation of deoxyribose. Similar to the reactions that occur in food, oxidation of endogenous polyunsaturated fatty acids has been proposed to generate furan via formation and cyclocondensation of 4-hydroxy-2-butenal (Onyango 2012). In contrast to 4-hydroxy-2-nonenal (HNE), a well-established endogenous lipid peroxidation product, endogenous generation of other 4-hydroxy-*trans*-2-alkenals such as 4-hydroxy-2-butenal has, however, not been demonstrated (Rietjens et al. 2022). In addition to lipid peroxidation, formation of *trans*-2-butene-1,4-dial by 5’-oxidation of deoxyribose has been suggested as a possible endogenous source of BDA-amino acid adducts (Chen et al. 2004), but experimental proof that this occurs at significant rates in mammalian cells and gives rise to the same type of adducts with GSH or lysine as *cis*-2-butene-1,4-dial (BDA) is still lacking. However, it is conceivable that *trans*-2-butene-1,4-dial formed from 5’-oxidation of deoxyribose may immediately react with close-by lysine residues, such as lysine residues on histones, which may be subsequently degraded to release Lys-BDA. The hypothesis that lysine residues on histones may be targeted by 2-butene-1,4-dial and its primary GSH-adduct is supported by identification of a cross-link between the GSH-BDA conjugate and lysine 107 of histone H2B in livers of rats treated with furan (Nunes et al. 2016). Based on the available data, it is presently not possible to conclude on the origin of the high background of BDA-derived lysine adducts and lysine-cysteine cross-links. As emphasized in a recent review, however, understanding the role of endogenous versus exogenous sources of process related food contaminants is critical for comprehensive exposure and risk assessment (Rietjens et al. 2022). In the case of furan-derived metabolites, it is evident that—if not considered in human biomonitoring studies—the high background levels of potentially endogenously formed metabolites excreted via urine may lead to an overestimation of furan exposure via food and consequently to an overestimation of the related human risk.

In contrast to NAcLys-BDA, NAcCys-BDA-NAcLys and NAcCys-BDA-NAcLys sulfoxide, which are thought to arise primarily from the reaction of BDA with protein-bound lysine and cysteine residues, GSH-BDA showed no background excretion. The absence of background levels and close correlation between external dose and 24 h excretion of GSH-[^13^C_4_]-BDA support GSH-BDA as a specific biomarker to monitor external exposure to furan. Excretion rates of GSH-[^13^C_4_]-BDA were low (< 2.5% within 24 h). This is, however, consistent with previous work demonstrating elimination of 20% of the orally ingested furan dose via urine in form of < 10 different metabolites (Burka et al. 1991), which arise from alkylation and cross-linking of GSH and free and protein-bound amino acids by the bifunctional electrophile BDA. The low excretion rates of GSH-BDA may prove difficult for human biomonitoring, in particular for translating human urinary biomarker concentrations into probable daily intakes, as minor differences in excretion rates such as those observed between male and female rats may have a significant impact on calculated intakes. Thus, studies in humans are now needed to test if biomonitoring of GSH-BDA is able to provide reliable exposure estimates. To this end, human toxicokinetic studies on furan similar to the present study in rats may be valuable to accurately determine GSH-BDA excretion rates in humans, also considering potential gender differences as suggested by our rat data.

In a recent study, elimination kinetics of the furan metabolites GSH-BDA, Lys-BDA and NAcLys-BDA and corresponding metabolites of 2-methylfuran were assessed in human volunteers after consumption of 500 mL of coffee brew containing a defined amount of furan and 2-methylfuran (Kremer et al. 2023). Participants were reported to eliminate 89.1 ± 21% of the ingested furan dose in urine within 24 h, with Lys-BDA accounting for 10.6 ± 4.4%, NAcLys-BDA accounting for 78 ± 18%, and consequently GSH-BDA accounting for less than 1% of the ingested dose (Kremer et al. 2023). While the low excretion rates of GSH-BDA are consistent with data in rats, the conclusion that in humans almost 90% of the furan dose are eliminated in urine is at odds with the present and previous studies in rats and extensive binding of furan to tissue proteins (Burka et al. 1991; Karlstetter and Mally 2020). Species-differences in renal vs. biliary excretion may contribute to this discrepancy. A possible alternative explanation is that background levels of NAcLys-BDA and Lys-BDA appear not to have been taken into account in the human study. This may lead to overestimation of excretion rates and consequently wrong estimates of human exposure when translating biomarker data into probable daily intakes. The overall conclusion that Lys-BDA and NAcLys-BDA may be suitable as short-term biomarkers of furan exposure (Kremer et al. 2023) is not supported by our present data. Interestingly, overall excretion rates of 2-methylfuran-derived metabolites were reported to be significantly lower (15.4 ± 4.8%) as compared to furan metabolites (89.1 ± 21%) (Kremer et al. 2023). Lys-AcA and NAcLys-AcA, i.e., two metabolites derived from acetyl acrolein, the reactive intermediate formed by cytochrome P450 dependent oxidation of 2-methylfuran, were also detected in human volunteers prior to coffee consumption. Whether this is due to intake of 2-methylfuran via food or potential endogenous formation is currently unclear. Although a pathway for formation of 2-methylfuran from omega-3 fatty acids via 2-hydroxy-2-pentenal has been proposed (Onyango 2012), there appears to be no evidence for potential endogenous formation of 2-methylfuran or its reactive metabolite acetyl acrolein so far. Thus, it remains to be established if Lys-AcA and AcLys-AcA provide reliable biomarkers to monitor exposure to 2-methylfuran. Controlled exposures in experimental animals in analogy to our present study may be valuable to understand if significant background levels of these metabolites occur.

Overall, our data support the use of GSH-BDA for monitoring furan exposure but highlight significant limitations of NAcLys-BDA, NAcCys-BDA-NAcLys and NAcCys-BDA-NAcLys sulfoxide due to their high background in urine. Future work is needed to confirm GSH-BDA as a specific biomarker of human exposure to furan via food and to assess if analysis of urinary GSH-BDA may provide reliable exposure estimates.

### Supplementary Information

Below is the link to the electronic supplementary material.

Supplementary file1 (DOCX 721 KB)

## Data Availability

All data supporting the findings of this study are available within the paper and its Supplementary Information. Raw data are available from the corresponding author upon reasonable request.

## Abbreviations

BDA: *cis*-2-Butene-1,4-dial
GSH: Glutathione
NAcLys: *N*-Acetyl-lysine
NAcCys: *N*-Acetyl-cysteine
GSH-BDA: *N*-[4-Carboxy-4-(3-mercapto-1*H*-pyrrol-1-yl)-1-oxobutyl]-l-cysteinylglycine
NAcLys-BDA: *R*-2-(Acetylamino)-6-(2,5-dihydro-2-oxo-1*H*-pyrrol-1-yl)-1-hexanoic acid
NAcCys-BDA-NAcLys: *N*-Acetyl-S-[1-[5-(acetylamino)-5-carboxypentyl]-1*H*-pyrrol-3-yl]-l-cysteine
NAcCys-BDA-NAcLys sulfoxide: *N*-Acetyl-S-[1-[5-(acetylamino)-5-carboxypentyl]-1*H*-pyrrol-3-yl]- l-cysteine sulfoxide
ESI–LC–MS/MS: Electrospray ionization liquid chromatography tandem mass spectrometry
HS–GC–MS: Headspace gas chromatography mass spectrometry
MRM: Multiple reaction monitoring
TIC: Total ion chromatogram
SIM: Selected ion monitoring

## References

[CR1] Burka LT, Washburn KD, Irwin RD (1991). Disposition of [14C]furan in the male F344 rat. J Toxicol Environ Health.

[CR2] Chen LJ, Hecht SS, Peterson LA (1997). Characterization of amino acid and glutathione adducts of cis-2-butene-1,4-dial, a reactive metabolite of furan. Chem Res Toxicol.

[CR3] Chen B, Bohnert T, Zhou X, Dedon PC (2004). 5‘-(2-Phosphoryl-1,4-dioxobutane) as a product of 5‘-oxidation of deoxyribose in DNA: elimination as trans-1,4-dioxo-2-butene and approaches to analysis. Chem Res Toxicol.

[CR4] European Food Safety Authority (2017). Risks for public health related to the presence of furan and methylfurans in food. Eur Food Saf Authority.

[CR5] Food and Drug Administration (2004) Determination of furan in foods

[CR6] Hamberger CMC, Kellert M, Schauer MDU, Dekant W, Mally A (2010). Hepatobiliary toxicity of furan: Identification of furan metabolites in bile of male F344/N rats. Drug Metab Dispos.

[CR7] Karlstetter D, Mally A (2020). Biomonitoring of heat-induced food contaminants: quantitative analysis of furan dependent glutathione- and lysine-adducts in rat urine as putative biomarkers of exposure. Food Chem Toxicol.

[CR8] Kellert M, Wagner S, Lutz U, Lutz WK (2008). Biomarkers of furan exposure by metabolic profiling of rat urine with liquid chromatography-tandem mass spectrometry and principal component analysis. Chem Res Toxicol.

[CR9] Kremer JI, Karlstetter D, Kirsch V, Bohlen D, Klier C, Rotermund J, Thomas H, Lang L, Becker H, Bakuradze T, Stegmüller S, Richling E (2023). Stable isotope dilution analysis (SIDA) to determine metabolites of furan and 2-methylfuran in human urine samples: a pilot study. Metabolites.

[CR10] Lachenmeier DW, Reusch H, Kuballa T (2009). Risk assessment of furan in commercially jarred baby foods, including insights into its occurrence and formation in freshly home-cooked foods for infants and young children. Food Addit Contam A.

[CR11] Lu D, Sullivan MM, Phillips MB, Peterson LA (2009). Degraded protein adducts of cis-2-butene-1,4-dial are urinary and hepatocyte metabolites of furan. Chem Res Toxicol.

[CR12] Nunes J, Martins IL, Charneira C, Pogribny IP, de Conti A, Beland FA, Marques MM, Jacob CC, Antunes AMM (2016). New insights into the molecular mechanisms of chemical carcinogenesis: In vivo adduction of histone H2B by a reactive metabolite of the chemical carcinogen furan. Toxicol Lett.

[CR13] Onyango AN (2012). Small reactive carbonyl compounds as tissue lipid oxidation products; and the mechanisms of their formation thereby. Chem Phys Lipid.

[CR14] Rietjens I, Dussort P, Günther H, Hanlon P, Honda H, Mally A, O’Hagan S, Scholz G, Seidel A, Swenberg J, Teeguarden J, Eisenbrand G (2018). Exposure assessment of process-related contaminants in food by biomarker monitoring. Arch Toxicol.

[CR15] Rietjens IMCM, Michael A, Bolt HM, Siméon B, Andrea H, Nils H, Christine K, Angela M, Gloria P, Daniel R, Natalie T, Gerhard E (2022). The role of endogenous versus exogenous sources in the exposome of putative genotoxins and consequences for risk assessment. Arch Toxicol.

