# Supplementary material for: Validation of putative biomarkers of furan exposure through quantitative analysis of furan metabolites in urine of F344 rats exposed to stable isotope labeled furan
Source: Arch Toxicol. 2024 Apr 4;98(6):1741–56. doi: 10.1007/s00204-024-03722-5 (PMC11106183; doi:10.1007/s00204-024-03722-5)
Supplement: Supplementary file 1 — Supplementary file1 (DOCX 721 KB) [file 204_2024_3722_MOESM1_ESM.docx]

**Supplementary material**

**Supplementary figures**

**Figure S1**

**Figure S1**: Enhanced product ion (EPI) scans of GSH-BDA (A), GSH-[^13^C_4_]-BDA (B) and [^13^C_2_^15^N]-GSH-BDA (C). The fragmentation pattern of GSH-BDA is consistent with literature data (Lu *et al.* 2009; Karlstetter and Mally, 2020).

**Figure S2**

**
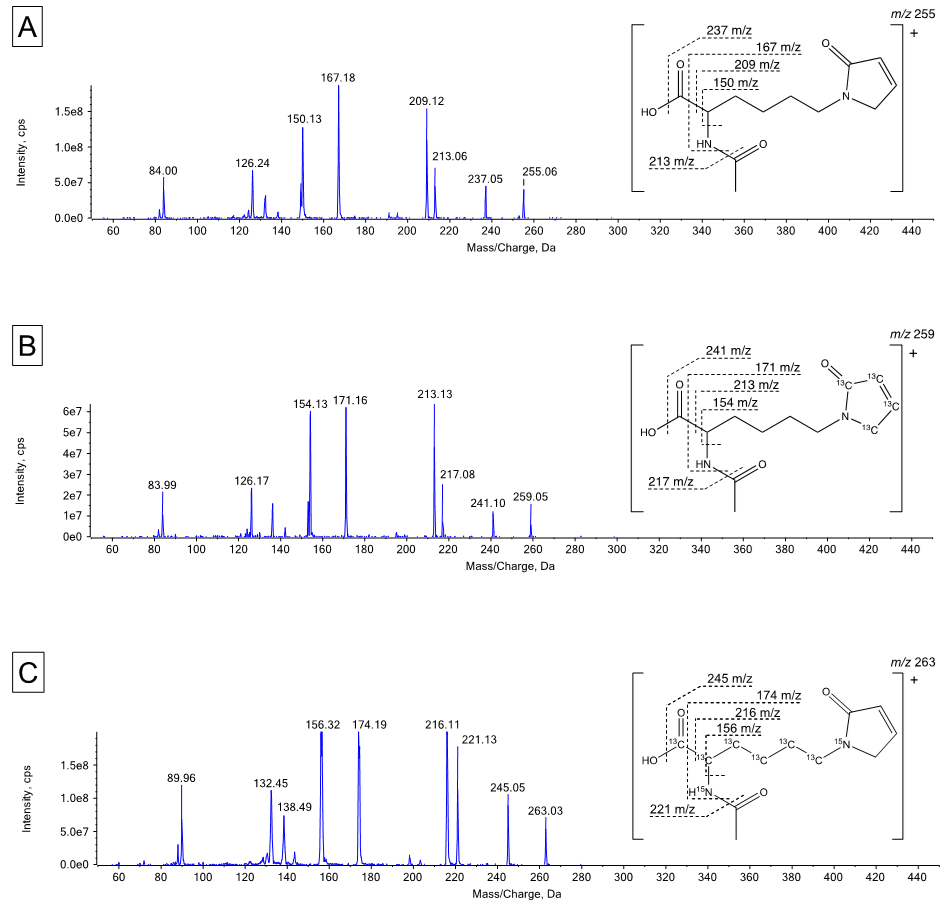
**

**Figure S2**: Enhanced product ion (EPI) scans of NAcLys-BDA (A), NAcLys-[^13^C_4_]-BDA (B) and NAc-[^13^C_6_^15^N_2_]-Lys-BDA (C). EPI spectra are consistent with previous reports (Chen *et al.* 1997; Karlstetter and Mally, 2020).

**Figure S3**

**Figure S3**: Enhanced product ion (EPI) scans of NAcCys-BDA-NAcLys (A), NAcCys-[^13^C_4_]-BDA-NAcLys (B) and [^13^C_2_]-NAcCys-BDA-NAcLys (C). The fragmentation pattern of NAcCys-BDA-NAcCys is consistent with previous reports (Lu *et al.* 2009).

**Figure S4**


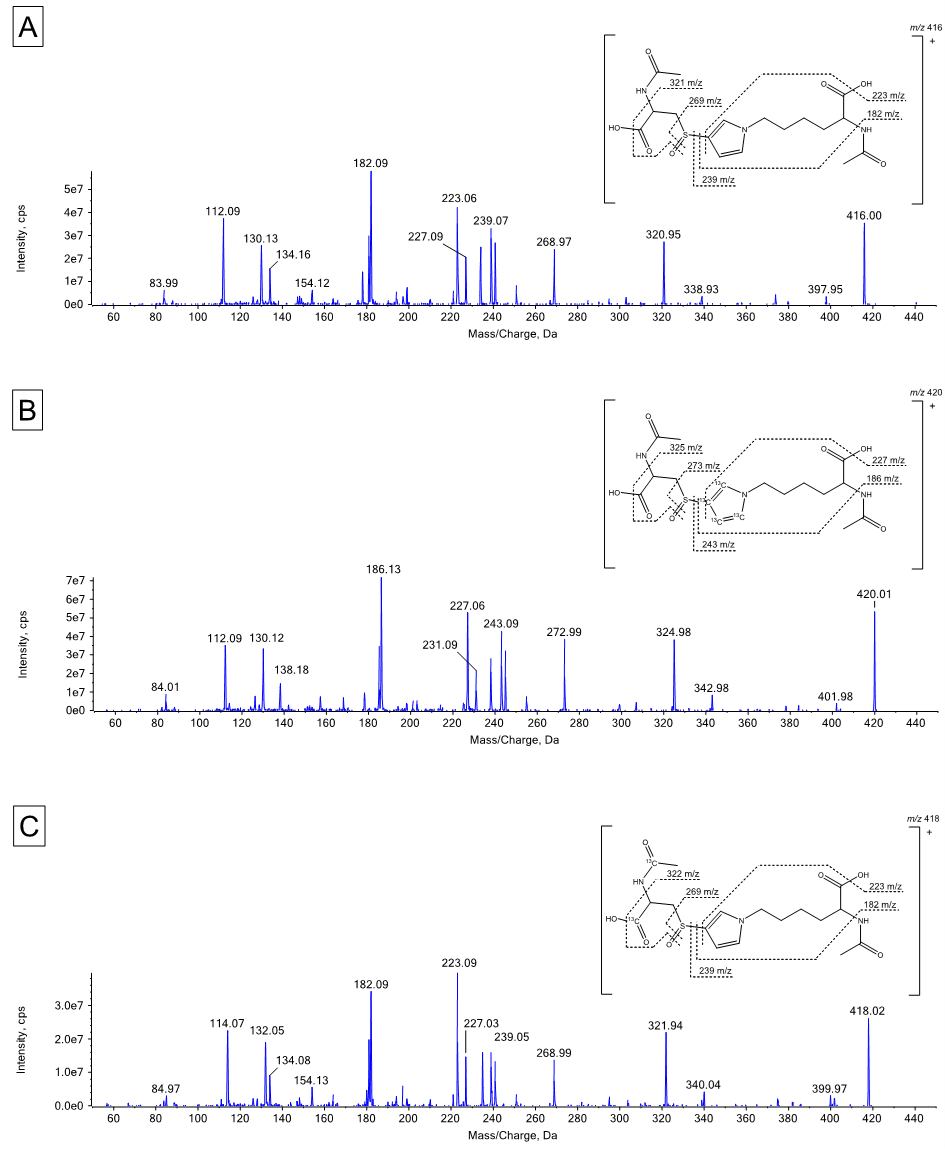


**Figure S4**: Enhanced product ion (EPI) scans of NAcCys-BDA-NAcCys sulfoxide (A), NAcCys-[^13^C_4_]-BDA-NAcCys sulfoxide (B) and [^13^C_2_]-NAcCys-BDA-NAcLys (C). EPIs of are consistent with literature data reported for NAcCys-BDA-NAcCys sulfoxide (Lu *et al.* 2009).

**Supplementary tables**

**Table S1:** Adjustment of actual vs. nominal dose of [^13^C_4_]-furan administered to male and female rats based on analysis of the [^13^C_4_]-furan content of the stock solutions via HS-GC-MS.

|  | **Stock solutions**  **[µg/mL]** | | **Dilution** | **Dosing solutions**  **[µg/mL]** | | **[^13^C_4_]-Furan dose**  **[µg/kg bw]** | | **[^13^C_4_]-Furan dose**  **[nmol/kg bw]** | |
| --- | --- | --- | --- | --- | --- | --- | --- | --- | --- |
|  | Nominal | Measured |  | Nominal | Actual | Nominal | Actual | Nominal | Actual |
| **male rats** | 450 | 634.5 | 1:1.8 | 250 | 353 | 1000 | 1410 | 13889 | 19583 |
|  |  |  | 1:18 | 25 | 35 | 100 | 141 | 1389 | 1958 |
|  |  |  | 1:180 | 2.5 | 3.5 | 10 | 14 | 139 | 196 |
|  | 50 | 55.9 | 1:200 | 0.25 | 0.3 | 1.0 | 1.1 | 14 | 16 |
|  |  |  | 1:2000 | 0.025 | 0.03 | 0.1 | 0.1 | 1,4 | 1.6 |
| **female rats** | 450 | 644.1 | 1:1.8 | 250 | 358 | 1000 | 1431 | 13889 | 19880 |
|  |  |  | 1:18 | 25 | 36 | 100 | 143 | 1389 | 1988 |
|  |  |  | 1:180 | 2.5 | 3.6 | 10 | 14 | 139 | 199 |
|  | 50 | 55.9 | 1:200 | 0.25 | 0.3 | 1.0 | 1.1 | 14 | 16 |
|  |  |  | 1:2000 | 0.025 | 0.03 | 0.1 | 0.1 | 1,4 | 1.6 |
